# Development of Monoclonal Antibodies Targeting Canine PD-L1 and PD-1 and Their Clinical Relevance in Canine Apocrine Gland Anal Sac Adenocarcinoma

**DOI:** 10.3390/cancers14246188

**Published:** 2022-12-14

**Authors:** Lucia Minoli, Luca Licenziato, Mikolaj Kocikowski, Marzia Cino, Katarzyna Dziubek, Selina Iussich, Antonella Fanelli, Emanuela Morello, Marina Martano, Ted Hupp, Borek Vojtesek, Maciej Parys, Luca Aresu

**Affiliations:** 1 Department of Veterinary Sciences, University of Turin, Largo Braccini 2, 10095 Grugliasco, Italy; 2 International Centre for Cancer Vaccine Science, University of Gdansk, Kladki 24, 80822 Gdansk, Poland; 3 Department of Veterinary Sciences, University of Parma, Strada del Taglio 10, 43100 Parma, Italy; 4 Institute of Genetics and Cancer, The University of Edinburgh, Crewe Road, Edinburgh EH4 2XU, UK; 5 Research Centre for Applied Molecular Oncology, Masaryk Memorial Cancer Institute, Zluty kopec 7, 65653 Brno, Czech Republic; 6 Royal (Dick) School of Veterinary Studies and Roslin Institute, The University of Edinburgh, Easter Bush Campus, Midlothian EH25 9RG, UK

**Keywords:** dog, apocrine gland anal sac adenocarcinoma, PD-1, PD-L1, tumor-infiltrating lymphocytes, immunohistochemistry

## Abstract

**Simple Summary:**

In human cancers, the development of PD-1 and PD-L1 inhibitors has dramatically increased survival in many patients, but only recently these molecules have been considered in veterinary medicine. Here, we describe generation of specific canine PD-1 and PD-L1 monoclonal antibodies and validation in canine apocrine gland anal sac adenocarcinoma (AGASACA), a previously identified tumor characterized by aberrant immune checkpoint activation. Both PD-1 and PD-L1 antibodies showed specificity for the canine ligand and functional activity in the in vitro assays. In the tumors, a variable PD-1 expression was detected in the intratumor and peritumor lymphocytes. Furthermore, 42% of AGASACA expressed PD-L1 and had a lower survival when treated with surgery alone. Taken together, these results demonstrate that the canine PD-1/PD-L1 axis is relevant in AGASACA and the inhibition might represent an effective strategy after surgery. Future experiments are ongoing to demonstrate the therapeutic potential of the generated monoclonal antibodies.

**Abstract:**

Canine apocrine gland anal sac adenocarcinoma (AGASACA) is an aggressive canine tumor originating from the anal sac glands. Surgical resection, with or without adjuvant chemotherapy, represents the standard of care for this tumor, but the outcome is generally poor, particularly for tumors diagnosed at an advanced stage. For this reason, novel treatment options are warranted, and a few recent reports have suggested the activation of the immune checkpoint axis in canine AGASACA. In our study, we developed canine-specific monoclonal antibodies targeting PD-1 and PD-L1. A total of 41 AGASACAs with complete clinical and follow-up information were then analyzed by immunohistochemistry for the expression of the two checkpoint molecules (PD-L1 and PD-1) and the presence of tumor-infiltrating lymphocytes (CD3 and CD20), which were evaluated within the tumor bulk (intratumor) and in the surrounding stroma (peritumor). Seventeen AGASACAs (42%) expressed PD-L1 in a range between 5% and 95%. The intratumor lymphocytes were predominantly CD3+ T-cells and were positively correlated with the number of PD-1+ intratumor lymphocytes (*ρ* = 0.36; *p =* 0.02). The peritumor lymphocytes were a mixture of CD3+ and CD20+ cells with variable PD-1 expression (range 0–50%). PD-L1 expression negatively affected survival only in the subgroup of dogs treated with surgery alone (*n =* 14; 576 vs. 235 days). The presence of a heterogeneous lymphocytic infiltrate and the expression of PD-1 and PD-L1 molecules support the relevance of the immune microenvironment in canine AGASACAs and the potential value of immune checkpoints as promising therapeutic targets.

## 1. Introduction

Apocrine gland anal sac adenocarcinoma (AGASACA) is a relatively rare skin neoplasm, representing approximately 17% of perianal tumors in dogs [1]. It is characterized by aggressive biological behavior, with a high rate of metastasis affecting the locoregional lymph nodes and, less frequently, distant sites. The median survival time ranges from one to two years, depending on the clinical stage [1,2,3,4,5,6,7,8,9,10,11,12,13]. The prognostic factors include regional lymph node metastasis at the time of diagnosis, tumor and regional lymph node size, histological pattern, necrosis, and lymphovascular invasion [14,15,16,17,18]. Conversely, the prognostic significance of hypercalcemia remains controversial, and the Ki67 proliferation index does not demonstrate prognostic value [18,19]. Surgical excision represents the best treatment option for AGASACAs without distant metastasis [2]. Chemotherapy and radiotherapy are also employed as adjuvant and/or palliative treatments, and toceranib phosphate showed effective results for prolonging survival time [3,4,5,6,7,8,9,10,11,12,13,20]. Immunotherapeutic approaches have not been explored yet.

In several canine cancers, the immune checkpoint programmed death-1 (PD-1) and its ligand PD-ligand 1 (PD-L1) were recently suggested as prognostic markers as well as promising therapeutic targets [21,22,23,24,25,26,27]. The interaction between PD-1 and PD-L1 is physiologically involved in the immune response regulation, resulting in the activation of inhibitory signals, and is responsible for the reduced production of antibodies and cytokines by the immune cells [28]. In oncology, the PD-1/PD-L1 axis activation induced by PD-L1-expressing tumor cells interacting with PD-1-expressing tumor-infiltrating lymphocytes (TILs) is one of the most studied immune evasion strategies played by cancer, paving the way for the development of immunotherapeutic approaches based on the blockade of these molecules [28]. Recently, the expression of PD-L1 was investigated by immunohistochemistry (IHC) in various canine tumors, among which 95% of the analyzed AGASACAs (19/20) expressed the immune checkpoint molecule, suggesting the activation of the axis [26]. However, these results were limited to a small number of cases, and the association with clinico-pathological data was not considered.

In this study, we developed canine-specific monoclonal antibodies targeting PD-1 and PD-L1. The expression of the two immune checkpoint molecules was evaluated by IHC in 41 surgically resected AGASACAs. The immunophenotypic characterization of the TILs was also performed. Furthermore, a correlation between the IHC results and clinico-pathological features and follow-up data was investigated in order to elucidate the prognostic role of the immune microenvironment and to provide new therapeutic insights.

## 2. Materials and Methods

### 2.1. Study Population

The AGASACA cases diagnosed and treated at the Veterinary Teaching Hospital (VTH) of the University of Turin, spanning period of 10 years (2011–2021), were considered for the study. Only dogs that underwent the surgical excision of the primary tumor and of regional lymph nodes when found enlarged on CT scan evaluations were included in the study, regardless of the administration of the adjuvant chemotherapy. Dogs diagnosed with stage IV disease before the surgery or having concurrent severe illnesses that could significantly reduce the survival time were excluded.

For each dog, signalment, primary tumor side, tumor size, clinical signs at presentation, presence of hypercalcemia (ionized calcium >1.45 mmol/L), presence of ileo-sacral lymphadenopathy, complete blood analysis, pre- and postcontrast whole body computed tomography, type of treatment (surgery with or without adjuvant chemotherapy), and surgical and postsurgical complications were recorded. Follow-up data were obtained from the follow-up visits at the VTH and/or by telephone contact with the owners or referring veterinarians. The disease-free interval (DFI) was calculated as the interval between surgery and local recurrence or metastatic spread, and the survival time (ST) was the time from surgery until death for any cause.

### 2.2. Histological Analysis

All surgically removed AGASACAs were histologically confirmed, and the following morphological features were recorded [19]: predominant tumor pattern (solid, rosettes/tubules, and papillary), necrosis (absent or present), inflammatory infiltration (absent or present), status of surgical margins, lympho-vascular invasion (absent or present), cellular pleomorphism (anisokaryosis and anisocytosis), mitotic count, and Ki67 index evaluated by IHC.

### 2.3. Generation of Anti-PD-1 and Anti-PD-L1 Monoclonal Antibodies

Anti-PD-1 and anti-PD-L1 monoclonal antibodies were generated by Moravian-Biotechnology Ltd. (Brno, Czech Republic). BALB/c mice were hyperimmunized with canine recombinant PD-1 and PD-L1 proteins (with a 6xHis and Fc tag, respectively; Sino Biological Europe GmbH, Eschborn, Germany). For both targets, splenocytes from hyper-immunized mice were fused with the nonproducing mouse myeloma cell line SP2. Supernatants of selected hybrids were screened using dot blot on a nitrocellulose membrane coated with recombinant canine PD1 (Sino Biological Europe GmbH, Eschborn, Germany) or PD-L1 (KingFisher Biotech Inc, Saint Paul, MN, USA) [29]. Reactive supernatants were further validated by ELISA, Western blotting, and flow cytometry.

### 2.4. ELISA–PD-L1

Recombinant PD-L1-FC fusion protein (Sino Biological Europe GmbH, Eschborn, Germany) was diluted in carbonate coating buffer (pH 9.6) at 1 µg/mL, and a 96-well plate (Costar) was coated with 50 ng of protein overnight at 4°C. Wells were subsequently washed three times with PBST and blocked with blocking buffer (PBS with 2% BSA) for 1 h at room temperature. Blocking buffer was removed, and serial dilutions of antibodies (1:50, 1:100, 1:200, 1:400, 1:800, and 1:1600—starting from 1 mg/mL) were added and incubated overnight at 4 °C. Wells were then emptied, washed three times with PBST, and incubated for 1 h at room temperature with goat antimouse HRP conjugated antibody (Dako) diluted in PBS with 2% BSA at 1:1000. After washing the wells, Pierce ECL (Thermo Fisher Scientific, Oxford, UK) was added to the wells and the plate was read using BioTek Synergy plate reader. Data were subsequently analyzed using GraphPad Prism 9.4 (GraphPad, San Diego, CA, USA).

### 2.5. ELISA–PD-1

Recombinant PD-1-FC fusion protein (Sino Biological) was diluted in carbonate coating buffer (in-house; pH 9.6). A 96-well opaque white plate (Costar) was coated overnight at 4 °C with 50ng of protein per well. Wells were subsequently washed four times with 400 μL of PBST 0,05% (the same for all the following washes) and blocked with blocking buffer (BB; PBST with 3% BSA) for 90 min at room temperature (RT). Blocking buffer was removed, and serial dilutions of the antibody (purified stock in PBS) were prepared (1000, 100, 10, 1 ng/mL, equal to 100, 10, 1, 0.1 ng/well) and added to a plate: 100 μL per well. The plate was incubated for 1h in RT. Wells were emptied, washed, and incubated for 1 h at room temperature with a rabbit antimouse polyclonal HRP conjugated antibody (Dako P0260) diluted in BB at 1:1000. After washing the wells, Pierce ECL (Thermo Fisher) was added to the wells and the plate was read immediately using a Fluoroskan Ascent plate reader (Thermo Fisher). Luminescence mode and integration time: 300ms. Data were analyzed using GraphPad Prism 9.4 (GraphPad, San Diego, CA, USA).

### 2.6. Western Blotting

Western blotting (WB) for PD-L1 analysis was performed using recombinant PD-L1- Fc fusion protein (Sino Biological Europe GmbH, Eschborn, Germany), with cell lysates of K9TCC, K9TCC-SH (both kindly provided by Prof. Deborah Knapp, Purdue University, USA), and DH82 (ATCC), K9TCC, K9TCC-SH, and DH82. All cell lines were selected based on previous publications, which have shown that the cell lines expressed PD-1 and/or PD-L1 [27,30,31]. In addition, the K9TCC and K9TCC-SH were considered to have different levels of expression of EGFR [32]. EGFR is known to affect PD-L1 stability through modifications in glycosylation levels, allowing to investigate antibody bindings to different patterns of PD-L1 glycosylation [33].

Thirty micrograms of cell lysates were separated on Novex NuPage 4–12% SDS-PAGE gel (Thermo Fisher Scientific, Oxford, UK) and transferred to PVDF membrane using iBlot transfer system (Thermo Fisher Scientific, Oxford, UK). Membranes were then blocked with Intercept^®®^ (TBS) Blocking Buffer (Li-cor, Cambridge, UK) and incubated with PD1 1.1 or PD-L1 3.1 antibodies at 1:1000 (PD-1 1.1) or 1:200 (PD-L1 3.1) concentration overnight. After the incubation, membranes were washed and incubated with IRDye^®®^ 800CW Donkey antimouse (Li-cor, Cambridge, UK). The membrane was subsequently washed with PBST and PBS. The membrane was imaged using Odyssey imaging system (Li-cor, Cambridge, UK).

### 2.7. Flow Cytometry

The cell lines used for WB were also utilized in flow cytometry (FC) experiments. Cells were expanded in DMEM/F12 (K9TCC, K9TCC SH) or DMEM (DH82, OSA31) media (both from Gibco, Thermo Fisher Scientific, Oxford, UK) supplemented with 10% fetal bovine serum (FBS) (Gibco, Thermo Fisher Scientific, Oxford, UK) and Penicillin-Streptomycin (Gibco, Thermo Fisher Scientific, Oxford UK). After reaching confluency, cells were detached using 0.25% Trypsin-EDTA (Gibco, Thermo Fisher Scientific, Oxford, UK), counted, and 5 × 10^5^ cells were used for each antibody. For all cell lines, PD-L1 antibodies 1.1, 2.1, and 3.1 were used at a concentration of 1:100 diluted in PBS with 2% BSA and incubated for 1 h. Subsequently, the cells were washed with PBS with 2% BSA and incubated with 1:1000 dilution of FITC conjugated antimouse polyvalent immunoglobulin secondary antibody (Sigma, Merck KGaA, Darmstadt, Germany), which binds all antibody subclasses. Cells incubated with secondary antibodies were used as controls. PD-1 antibody was directly conjugated to APC fluorophore using a commercially available kit (Abcam, Cambridge, UK) and did not require a secondary antibody. Unstained cells were used as controls for PD-1 antibody.

For the DH82 cell line, as it expresses Fc receptor, the cells were incubated with Fc Receptor Binding Inhibitor (eBioscience, ThermoFisher Scientific, Oxford, UK) for 30 min. Cells were then washed, resuspended in staining buffer, and flow cytometry was performed using BD LSR Fortessa flow cytometer (Becton Dickinson, Wokingham, UK). Data were subsequently analyzed using FlowJo (FlowJo LLC, Ashland, OR, USA).

In addition, a K9TCC transiently overexpressing PD-1 was generated via transfection of this cell line with a custom-build pcDNA3.1 plasmid vector encoding for canine PD-1 (GeneArt, ThermoFisher Scientific, Oxford, UK). Cells were transfected with the vector using lipofectamine (Gibco, ThermoFisher Scientific, Oxford, UK) and selected using Hygromycin (ThermoFisher Scientific, Oxford, UK). Transfection was confirmed using flow cytometry as described above.

### 2.8. Immunohistochemistry

Immunohistochemistry (IHC) was performed on 4 μm-thick FFPE sections using VECTASTAIN^®®^ Universal Quick HRP Kit, Peroxidase, RTU, from Vector Laboratories Inc. (Burlingame, CA, USA), following the manufacturer’s instructions. Sections were incubated with 0.3% H_2_O_2_ for endogenous peroxidase activity blocking, and heat-induced antigen retrieval was then performed in citrate buffer (pH 6.0) (for anti-PD-L1, anti-PD-1, anti-CD20 primary antibodies) or in Tris-EDTA buffer (pH 9.0) (for anti-CD3 primary antibody) at 98 °C for 20 min. After the nonspecific antibody binding blocking performed with Normal Horse Serum 2.5%, the sections were incubated with the following primary antibodies: anti-CD3 (diluted 1:50; Dako, Nowy Sącz, Poland), anti-CD20 (diluted 1:1000; Moravian-Biotechnology), and anti-PD-1 (diluted 1:100) for 2h at room temperature, and anti-PD-L1 (diluted 1:10) overnight at 4 °C. Signal was detected using Vectastain Elite ABC kit and ImmPACT DAB from Vector Laboratories Inc. (Burlingame, CA, USA). In each immunohistochemical run, the positive controls were included (canine lymph node for anti-CD3 and anti-CD20; canine tonsil for anti-PD-1; canine diffuse large B-cell lymphoma, known to have high PD-L1 mRNA expression by RNAscope for anti-PD-L1). Negative controls were prepared by replacing the primary antibody with an irrelevant one in all cases.

### 2.9. Immunohistochemical Scoring

IHC stained slides were independently evaluated by 2 pathologists (LM and LL), and the discordant results were reviewed to reach a consensus.

TILs were evaluated on the CD3- and CD20-stained slides and separately scored within the tumor bulk (i.e., intratumor lymphocytes) and in the surrounding stroma (i.e., peritumor lymphocytes). Briefly, intratumor lymphocytes were counted in 4 microscopic fields at 200x, and a score was arbitrarily assigned according to the following criteria: score 0 = < 10 positive lymphocytes; 1 = 10–25 positive lymphocytes; 2 = 26–60 positive lymphocytes; 3 = > 60 positive lymphocytes. Peritumor lymphocytes were scored from 0–3 according to the number of lymphocytic aggregates in the tumor stroma, regardless of their immunophenotype and counted in 10 fields at 4× magnification (“peritumor lymphocyte total score”; score 0 = ≤ 1 aggregates; 1 = 2–6 aggregates; 2 = 7–14 aggregates; 3 = ≥ 15 aggregates). In addition, the proportion of CD3 and CD20 positive lymphocytes in peritumor aggregates was recorded.

Similarly, PD-1-expressing lymphocytes were separately scored in intratumor and peritumor areas. For intratumor PD-1+ lymphocytes, the same scoring system described above for CD3 and CD20 was applied. For peritumor lymphocytes, the percentage of PD-1+ cells was assessed, and a semiquantitative score was assigned as follows: 0 = <5%; 1 = 5–24%; 2 = 25–49%; 3 = ≥50%.

For PD-L1 evaluation, the percentage and the intensity (0 = negative; 1 = mild; 2 = moderate; 3 = marked) of the positive tumor cells was assessed.

### 2.10. Statistical Analysis

All statistical analyses were performed using R software. The association of clinico-pathological features with the IHC results was explored. Fisher’s exact test was used for categorical variables, while continuous variables were tested by means of Student t-test or Wilcoxon rank-sum test, according to the results of a Shapiro–Wilk test previously performed to assess the normality of the distribution. The correlation between the IHC results was investigated by Spearman rank correlation testing. Survival analysis was conducted using *survival* and *survminer* packages. A univariate Cox proportional-hazards model was used to test the impact of the following variables on DFI and ST: age, sex, breed, weight, presence of lymph nodes metastasis at the time of the diagnosis, presence of hypercalcemia, tumor size (major diameter measured after formalin fixation), type of treatment (adjuvant chemotherapy in addition to surgery), local recurrence, occurrence of regional or distant metastasis, histopathological features, and IHC results. A cut-off of I ≤ 0.05 was used to screen the variables to be included in the multivariate analysis. Kaplan–Meier curves for the categorical variables were drawn and compared by means of log-rank testing.

## 3. Results

### 3.1. Study Population

A total of 41 dogs were included in the study (Table S1). Twenty-three (56.1%) were purebred dogs, including Labrador Retrievers (*n =* 9), Border Collies (*n =* 3), Czechoslvakian Wolfdogs (*n =* 3), and German Shepherds (*n =* 2). Thirty-two (78%) dogs were female (5 intact; 27 spayed), and 9 (22%) were male (4 intact; 5 neutered). The median age at diagnosis was 11 years (range: 5–15 years), and the median body weight was 24 kg (range: 7–40 kg).

Fifteen dogs (36.6%) were hypercalcemic at presentation, and three (7.3%) had a bilateral tumor mass. The median tumor size (major diameter measured after formalin fixation) was 3.5 cm (range: 0.5–10 cm). Thirty-one (75.6%) dogs had sublumbar lymphadenopathy at presentation, confirmed as metastatic AGASACA by histopathology in all cases. Fourteen dogs (34.1%) were treated only by surgical resection of the primary tumor and the regional lymph nodes. Twenty-seven dogs (65.8%) received adjuvant chemotherapy, which consisted of toceranib phosphate (*n =* 25), carboplatin (*n =* 1), and melphalan (*n =* 1).

### 3.2. Generation and Validation of Anti-PD-1 and Anti-PD-L1 Monoclonal Antibodies

For the PD-L1 antibodies, three clones were considered for further validation as they detected PD-L1-FC recombinant protein in the dot blot. The clones were called 1.1 (IgG3), 2.1 (IgG2a), and 3.1 (IgM). All three clones detected PD-L1-FC in ELISA (Figure S1A–C) but none were detected in the FC assay against cells constitutively expressing PD-L1 (Figure S1D–F, representing the 3.1 clone). In a WB against PD-L1-FC fusion protein, only one clone (IgM PD-L1 3.1) reacted, and a single band of approximately 56 kDa was detected (Figure S2 A). Hence, only the IgM PD-L1 3.1 clone was used for further analysis and tested in WB against the three canine cell lines (K9TCC, K9TCC-SH, and DH82), producing a dominating band of approximately 34 kDa and some weaker bands above it, likely representing the different post-translational forms of PD-L1 [34] (Figure S2B).

For the PD-1 antibodies, one clone, designated 1.1 (IgG2a), was chosen, which detected rcPD-1-His in the dot blot. PD-1 1.1 detected PD-1-FC in ELISA in a dose-dependent manner, within a range of 0.1–100 ng/well (Figure S3A). When the antibody was tested by flow cytometry against the K9TCC and K9TCC SH cells, constitutively expressing canine PD-1, the positive cells were identified (Figure S3B,C). In addition, an increased fluorescent signal was detected in the K9TCC cells overexpressing PD-1 (K9TCC-PD1 OE: Figure S3D). In WB, the antibody produced a dominant band, approximately 70 kDa in size, with additional, weaker bands underneath (Figure S4). Similar to PD-L1, PD-1 was heavily glycosylated. Interestingly, in the DH82 cell line, a single, weaker band at approximately 36 kDa was found, which is close to the size of deglycosylated PD-1 (Figure S4).

### 3.3. Histopathology and Immunohistochemistry

The histopathological features and IHC scores for each dog are reported in Table S1. The TILs were evaluated by IHC for CD3 (T lymphocytes) and CD20 (B lymphocytes), in addition to the immune checkpoint molecule PD-1.

**Figure 1:**
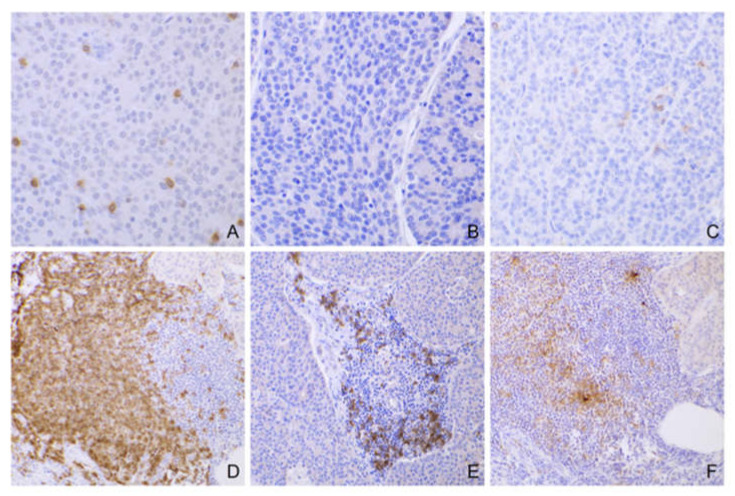
Tumor-infiltrating lymphocytes in canine AGASACAs. (**A**) Numerous intratumor CD3+ lymphocytes (case #22; 40× magnification). (**B**) Absence of intratumor CD20+ lymphocytes (case #22; 40× magnification). (**C**) Scattered intratumor PD-1+ lymphocytes (case #22; 40× magnification). (**D**–**F**) Peritumor lymphocytes in nodular aggregates are composed of a mixture of CD3 (**D**), CD20, (**E**) and PD-1 (**F**) positive lymphocytes (case #5; 20× magnification).

Intratumor lymphocytes were interspersed with the tumor cells in the tumor bulk and were predominantly CD3+ T cells, present in variable numbers: 2 cases scored 0; 15 cases scored 1; 14 cases scored 2; 10 cases scored 3 (Figure 1A). CD20+ intratumor lymphocytes were rarely detected (all cases scored 0) (Figure 1B). In 13 cases (31.7%), the intratumor lymphocytes also expressed PD-1 in a low percentage (score = 1), and a positive correlation between the number of CD3+ and PD-1+ intratumor lymphocytes was detected (*ρ* = 0.36; *p =* 0.02) (Figure 1C).

Regarding the peritumor lymphocytes total score, 1 case scored 0, 11 cases scored 1, 20 cases scored 2, and 9 cases scored 3. According to IHC, the peritumor lymphocytes were a mixture of CD3+ and CD20+ cells, often distributed in nodular aggregates, composed of a variable proportion of T and B lymphocytes (Figure 1D,E). Generally, the CD3+ cells predominated over the CD20+ cells, with a CD3+/CD20+ cell ratio ≥ 1 in 98% (40/41) of the cases. In 61% (25/41) of the cases, a variable proportion of peritumor lymphocytes also expressed PD-1 (11 with score 2; 14 with score 3) (Figure 1F). Local recurrence was significantly associated with a low peritumor lymphocyte total score (*p* = 0.009) and a low CD3+/CD20+ ratio in the peritumor lymphocytes (*p =* 0.034).

**Figure 2:**
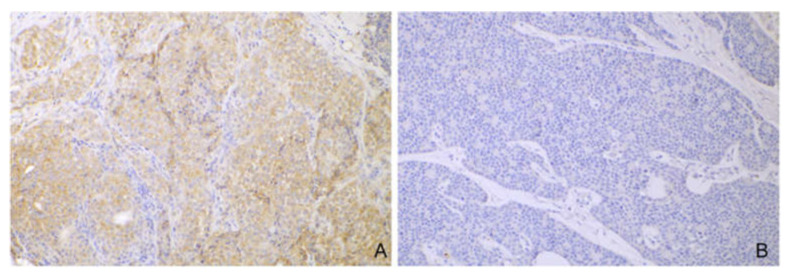
PD-L1 expression in canine AGASACAs (20× magnification). (**A**) Representative case with diffuse and moderate expression of PD-L1 (case #18). (**B**) Negative case (case #20).

Seventeen AGASACAs (42%) expressed PD-L1 in a range between 5% and 95% of the tumor cells, with mild to moderate intensity (Figure 2). Interestingly, a single case (case #36) was characterized by a regional area of undifferentiated tumor cells, displaying highly infiltrative behavior, spindeloid morphology, and increased cellular atypia. These cells showed diffuse and intense expression of PD-L1. No associations between PD-L1 expression and other IHC scores were identified.

### 3.4. Survival Analysis

At the time of the data analysis, five dogs (12.2%) were alive after 39–819 days, 22 (53.7%) had died for causes attributable to AGASACA (range: 128–1237 days), 14 (34.1%) for unrelated causes (range: 108–1434 days), and one was lost to follow-up (598 days). Eight dogs (19.5%) experienced metastatic disease to regional (iliosacral) lymph nodes after the primary surgery, while 11 dogs (26.8%) developed distant metastasis to various organs, including distant lymph nodes, spleen, liver, and lungs. The median DFI of the whole population was 474 days (range: 0–1237 days), and the median ST was 472 days (range: 108–1434 days). By univariate Cox proportional-hazard analysis, the dogs with metastatic regional lymph nodes at presentation had a significantly shorter DFI (369 vs. 940 days; *p =* 0.046) (Table S2). Body weight, local recurrence, and histological presence of lymphovascular invasion and tumor necrosis negatively affected the ST; however, only body weight and lymphovascular invasion maintained statistical significance in the multivariate analysis (*p =* 0.027 and *p =* 0.012, respectively) (Table S2).

**Figure 3:**
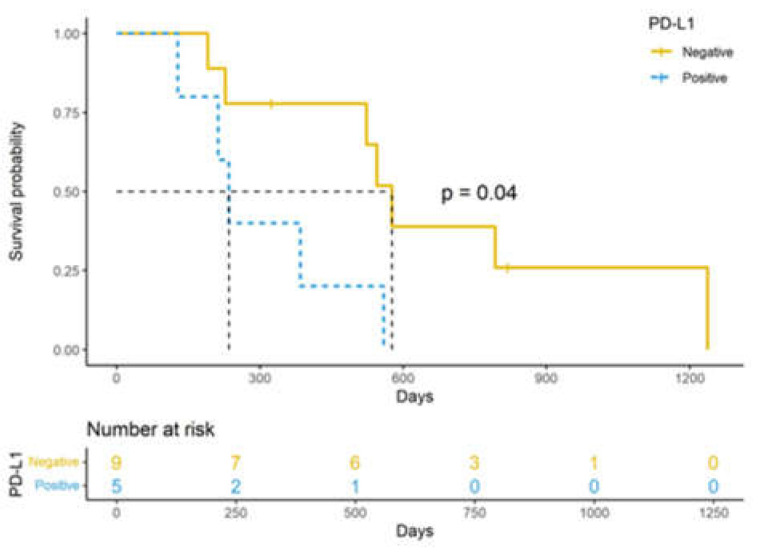
Kaplan–Meier curves of survival time of 14 dogs with AGASACA treated with surgery alone.

The TILs and immune checkpoint molecule expression did not influence the outcome in the whole population. When the subgroup of dogs treated by surgery alone (*n =* 14) was considered, tumor expression of PD-L1 was associated with a significantly shorter ST compared to cases that tested negative for the marker (576 vs. 235 days; *p =* 0.022) (Figure 3, Table S2).

## 4. Discussion

Deciphering the interaction between the tumor and the patient’s own immune system has led to the development of cancer immunotherapies, which recently revolutionized the paradigm of cancer treatment in human oncology. In this context, adaptative immunity, and intratumor lymphocytes, in particular, were shown to play a key role in the antitumor immune response [35]. Based on the degree of lymphocytic infiltration and molecular signatures, tumors can be classified into three different phenotypes (inflamed, immune-excluded, and immune-desert) that are strongly associated with the response to immunotherapeutic treatments, with inflamed (hot) tumors being more likely to benefit from the administration of checkpoint inhibitors [36]. Given the recognized role of several canine tumor types as a spontaneous model for human oncology, there is an increasing interest in characterizing the immune landscape of these tumors. Osteosarcoma, oral malignant melanoma, lymphoma, and mammary carcinomas belong to canine tumors presenting a hot immune phenotype [22,37].

To the best of our knowledge, the immune microenvironment of AGASACA has not been fully characterized so far, but a recent study suggested a possible association (even if not statistically significant) between inflammatory cell infiltration and outcome [19]. In addition, transcriptome analysis confirmed a “hot” immune signature [38].

Here, in order to further characterize AGASACA at the molecular level, we have developed two new antibodies against canine ICs that are highly relevant to cancer in both humans and dogs: PD-1 and PD-L1. The preliminary validation revealed that the PD-1 monoclonal antibody detects its target in WB, ELISA, and FC experiments. Among the three developed PD-L1 clones, all were effective in ELISA, none were effective in FC, and only the 3.1 clone was effective in WB. Upon basic validation, the antibodies PD-L1 3.1 and PD-1 1.1 were used to assess target expression on canine AGASACA samples. The antibodies will be further characterized in the future to assess their potential as drug candidates.

In the present study, the presence and phenotype of TILs were evaluated by IHC in 41 surgically resected AGASACAs, and a high degree of lymphocytic infiltration, both in peritumoral and intratumor locations, was observed. Moreover, while the peritumor lymphocytes were a mixture of T- and B-cells, the intratumor lymphocytes were almost exclusively represented by CD3+ T-cells. These findings support an inflamed immunophenotype for at least a subset of cases; in fact, contrary to the immune-excluded phenotype, which is characterized by lymphocytic infiltrates that are limited to the tumor stroma, inflamed tumors are defined by the presence of T-cells intermingled with neoplastic cells [36]. Even if the molecular investigations were beyond the aim of our study, the immunohistochemical characterization of the TILs suggests a “hot” phenotype for canine AGASACAs, and this encourages further investigation in this direction.

Cancer immunotherapy in veterinary oncology is still in the early stages of development and application. Some successful examples are represented by the clinical implementation of autologous vaccines in canine lymphoma [39] and anti-CSPG4 electrovaccination in canine melanoma [40]. Another early but promising example is represented by the empty cowpea mosaic virus treatment in inflammatory mammary carcinoma [41]. Only a few preliminary studies (by using specific canine anti-PD-L1 and anti-PD-1 antibodies for the treatment of oral malignant melanoma) demonstrated the potential value of immune checkpoint blockades in dogs [24,26]. In order to evaluate if canine AGASACA is a possible candidate for immunotherapy, the study population was tested by IHC with monoclonal antibodies specifically targeting canine PD-1 and PD-L1 and a subset of TILs expressing PD-1 in variable proportions. These were localized both within the tumor bulk (i.e., intratumor) and in the surrounding stroma (i.e., peritumor); moreover, in the intratumor areas, the number of PD-1+ lymphocytes positively correlated with the number of CD3+ cells. Although no prognostic value of PD-1 expression by TILs was demonstrated, the enrichment of the tumor microenvironment with PD-1-positive lymphocytes might support their role as a possible target for the development of immunotherapeutic strategies.

To date, the expression of PD-L1 at the protein level has been investigated in a variety of canine tumors. The most comprehensive studies, which included preliminary clinical trials or PD-L1 association with clinico-pathological features and/or outcomes, focused on melanoma, osteosarcoma, and mammary carcinoma [21,25,26,42]. In addition, two studies [26,43] tested the expression of PD-L1 by IHC in a limited number but a wide range of canine tumors, demonstrating its expression in several tumor types, including 19 out of 20 of the tested AGASACAs. Among these, most of the tumors expressed PD-L1 in more than 50% of the tumor cells. Similarly, in the present study, the expression of PD-L1 by 17 out of 41 AGASACAs was demonstrated, with variable percentages of positive tumor cells. A lack of standardized methods to assess PD-L1 expression by tumor cells in veterinary medicine might have contributed to the lower proportion of positive AGASACAs observed in our study compared to the previous one [26]. In this context, it is also worth mentioning the variability observed in human oncology where different antibody clones and staining platforms are used to assess the PD-L1 tumor proportional score [44]. Interestingly, a single case in this study had a regional tumor area displaying morphological features of undifferentiated carcinoma with a highly invasive growth pattern; this area was also characterized by diffuse and intense positivity for PD-L1. Given the morphological features and the early recurrence and regional metastasis observed in this dog, this finding might suggest an association between PD-L1 activation and aggressive tumor behavior.

The survival analysis for the whole cohort showed that body weight, local recurrence, and histological presence of lymphovascular invasion and tumor necrosis negatively affected the outcome, as previously described [18,19]. A shorter DFI was also observed in dogs with nodal metastasis at presentation.

PD-L1 expression was associated with a poorer outcome only in the subgroup of dogs treated with surgery alone (i.e., without medical treatment). This result indicates that similar to other canine tumors [21,23], PD-L1 expression in AGASACAs influenced tumor behavior, affecting the ST, thus representing a potential prognostic biomarker. However, due to the limited number of patients, this preliminary result needs to be interpreted with the consideration of potential bias and limitations. Future studies on a larger cohort of patients to confirm this result are needed. The lack of association between PD-L1 expression and survival when the whole cohort was considered might be explained by the influence that adjuvant treatment with toceranib phosphate has on survival [4,12,13]. In our study, we did not observe a clear impact of chemotherapy treatment on survival, but since the chemotherapy protocol was not standardized, it is possible that the adjuvant treatment might have represented a confounding factor when the whole population was considered, potentially obscuring the impact of other variables on the outcome. To our knowledge, the direct effect of toceranib phosphate on PD-L1 expression in canine cancer has not been investigated so far. However, there is some evidence for the induction of PD-L1 destabilization and degradation by sunitinib, a tyrosine kinase inhibitor similar to toceranib, leading to the decreased expression of this molecule on tumor cells [45]. Although not yet confirmed, the effect of toceranib might be similar.

## 5. Conclusions

In conclusion, our study showed the high and heterogenous expression of immune checkpoint molecules and the presence of lymphocytic infiltration in canine AGASACAs. PD-L1 was also associated with the outcome in a subset of patients, indicating the prognostic significance of this marker. Even if, in our cohort, the TILs were not associated with outcome, the high level of lymphocytic infiltration suggests a hot immune phenotype for this tumor. When taken together, these results show the relevance of the immune microenvironment in canine AGASACAs, indicating that the stimulation of anticancer immunity might represent a valuable treatment approach for this tumor type, and, as such, it deserves more in-depth investigations.

## Data Availability

Data is contained within the article and Supplementary Material.

## Supplementary Materials

The following supporting information can be downloaded at: https://www.mdpi.com/article/10.3390/cancers14246188/s1, Figure S1: PD-L1 antibody validation: ELISA and FC; Figure S2: PD-L1 antibody validation: Western Blot; Figure S3: PD-1 antibody validation: ELISA and FC; Figure S4: PD-1 antibody validation: Western Blot; Table S1: Clinicopathological features; Table S2: Survival.
