# Supplementary figures and images for: Development of Monoclonal Antibodies Targeting Canine PD-L1 and PD-1 and Their Clinical Relevance in Canine Apocrine Gland Anal Sac Adenocarcinoma
Source: Cancers (Basel). 2022 Dec 14;14(24):6188. doi: 10.3390/cancers14246188 (PMC9777308; doi:10.3390/cancers14246188)

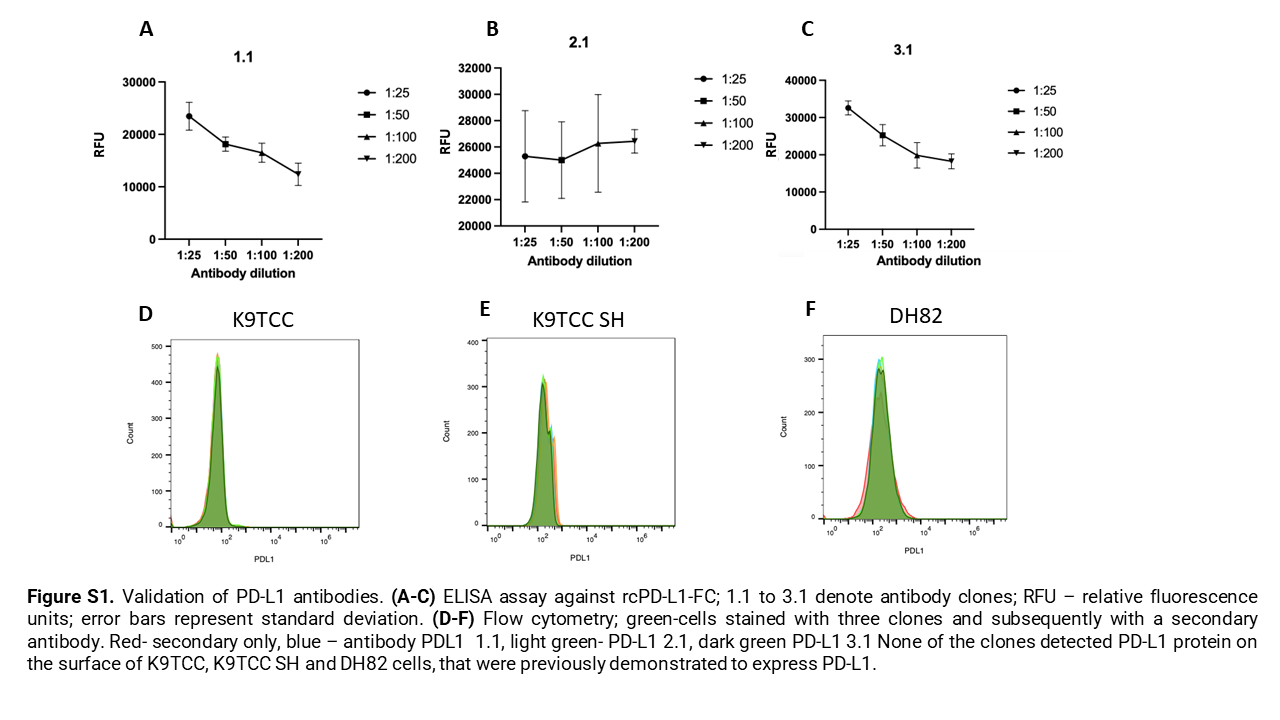

Supplement: Supplementary file 1 [file cancers-14-06188-s001.zip › Figure S1.TIF]

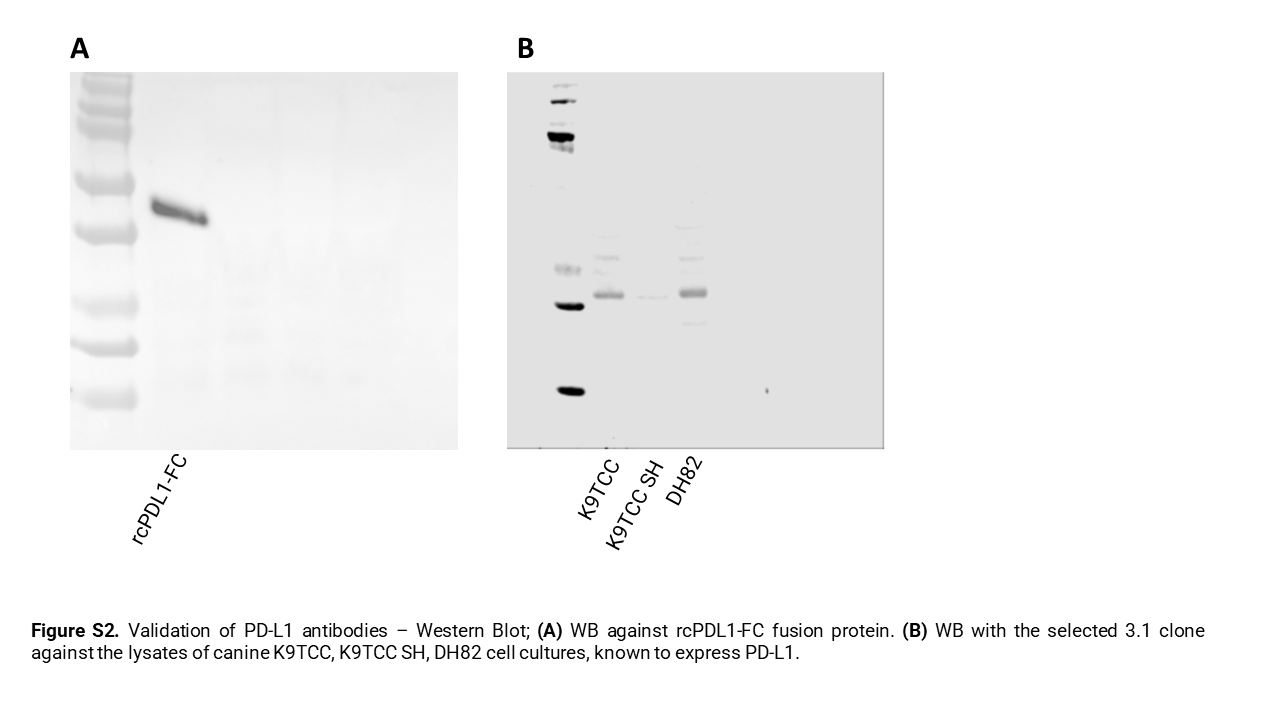

Supplement: Supplementary file 1 [file cancers-14-06188-s001.zip › Figure S2.TIF]

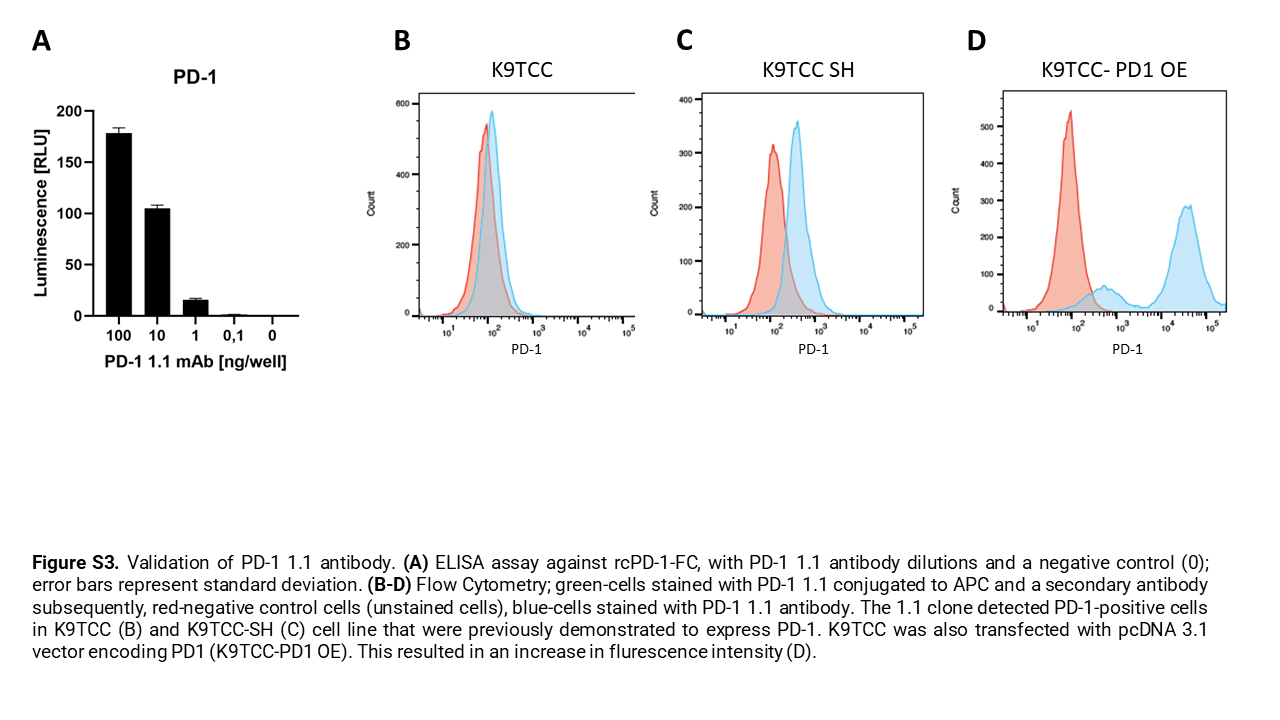

Supplement: Supplementary file 1 [file cancers-14-06188-s001.zip › Figure S3.TIF]

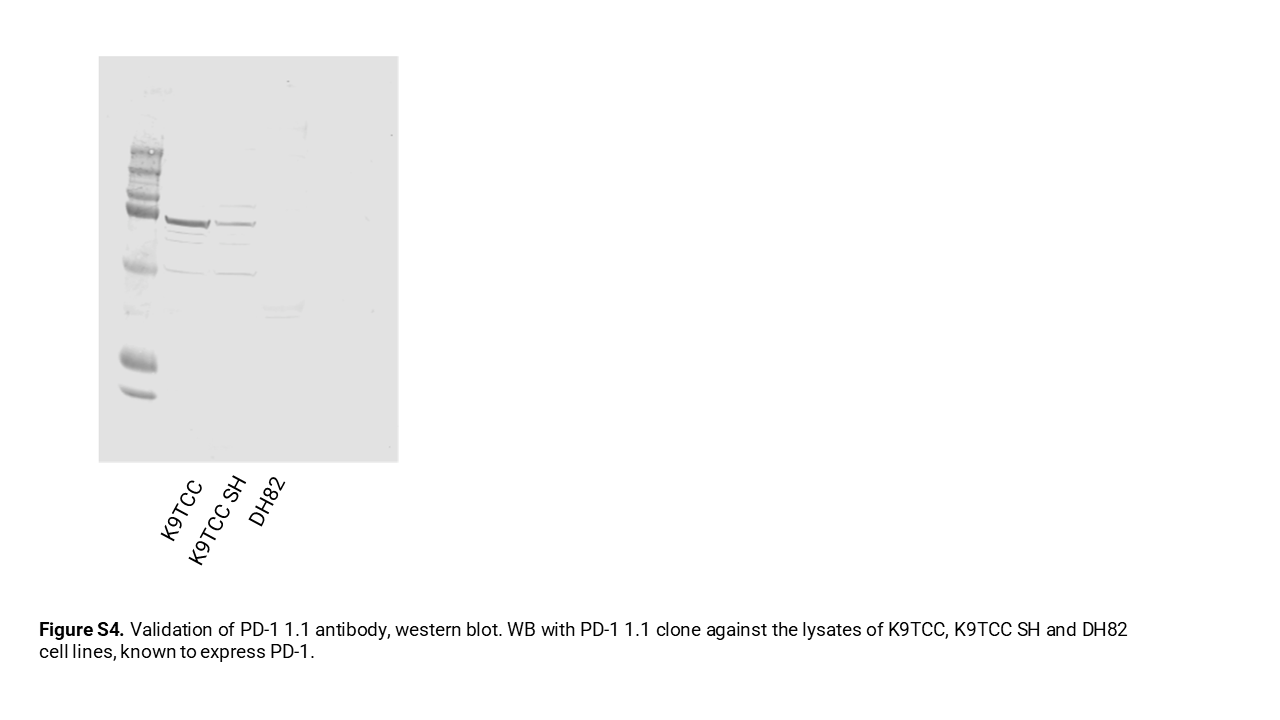

Supplement: Supplementary file 1 [file cancers-14-06188-s001.zip › Figure S4.TIF]
